# Hematological changes of silver carp (*hypophthalmichthys molitrix*) in response to Diazinon pesticide

**DOI:** 10.1186/s40201-015-0208-9

**Published:** 2015-06-16

**Authors:** Aliakbar Hedayati, Elaheh Hassan Nataj Niazie

**Affiliations:** Department of Fisheries, Faculty of Fisheries and Environment, Gorgan University of Agricultural Sciences and Natural Resources, Gorgan, Iran

**Keywords:** Blood indices, Diazinon, Fish, Pollution

## Abstract

Diazinon is widely consumed for plague control in the agricultural farms and in domestic and aquaculture aspects. The present research purposed to evaluate the effect of half-lethal concentration (LC50) of diazinon on some biochemical and hematological parameters of silver carp, *hypophthalmichthys molitrix*, after 0, 24, 48 and 96 h. The results showed that the values of leukocytes (WBC), haematocrit (Ht), hemoglobin (Hb), MCHC, lymphocyte, cortisol and glucose were significantly increased (*P* < 0.05). The mount of MCV and MCH were significantly increased at 48 h and then decreased at 96 h (*P* < 0.05). Moreover, there was a significant increasing in neutrophils count at 48 h, and then a significant decreasing at 96 h (*P* < 0.05). There were no significant differences in RBC, monocyte and eosinophile counts among treatment groups at different sampling intervals. Thereupon, low-term (96 h) exposure to diazinon at half-lethal concentration (LC50) caused biochemical and hematological changes in silver carp, and offers a simply implement to appraise toxicity-derivatived changes.

## Introduction

Diazinon is a vast spectrum OP, often utilized in residential areas, while its major utilization is with regard to agricultural activities. It has been categorized as very toxic and its use has been prohibited in many developed countries, however, it is commercialized in expansing ones [1]. Diazinon is partly water-soluble, and simply comes into contiguity with aquatic organisms. Afterwards, inward the cells of these organisms, diazinon is metabolized to diazoxon, which is the toxic composition of this insecticide. In addition, diazinon affects abroad range of non-target organisms, such as invertebrates, birds, mammals and fishes, chiefly, those are habiting aquatic environment [2]. Kalender et al. [3] showed that diazinon causes alters in liver enzymes and biochemical indicators. Diazinon is widely utilized in the rice paddy farms of Mazandaran, Gillan, Golestan and other areas in Iran [4]. Based on reports of Bulletin of Agriculture Ministry, the annual utilization of diazinon in iran is appraised to be 3775 t [5]. Several studies related that some of the surface waters and the surrounding environments in Iran were infected with organophosphate pesticides like diazinon and its derivates [6].

Hematological parameters like MCH, MCV and MCHC and Hematological indices such as Hct, Hb, RBC, WBC values and biochemical indicators like glucose are widely utilized to assess the toxic stress of environmental contaminants [7]. Silver carp may be most abundant freshwater fish due to its fast growth [8]. In spite of the manufacture of silver carp is spreading out annually, the processing of this fish is limited. Silver carp rearing developed in many ponds that placed near agricultural fields.

The aim of this study was to investigate the effect of different diazinon concentrations on blood biochemical, hematological and hormonal indicators in silver carp (*hypophthalmichthys molitrix*).

## Materials and methods

### Preparation of fish in experimental condition

Fish with average weight 200 g of silver carp after 1-week adaptation to the new condition, were divided into 4 treatments (3 replicates for each treatment) and a control with 21 fish in each 400 L tank. All fish were hand-fed with trading pellet twice a day. Fish were exposed to a concentrations of 50 % LC_50_ (3.93 ± 0.34) of diazinon for a period of 24, 48 and 96 h. Temperature, dissolved oxygen, conductivity and pH were measured during the experiment.

### Blood sampling and hematological assay

At the beginning, fish were unconscious with 200 ppm clove powder. Blood samples quickly collected from tail blood vessel by heparinized syringes and immediately stored on ice. Then, computation of the blood parameters were carried out on fresh blood. Numbers of Blood erythrocytes and leukocytes were performed by diluting heparinized blood with Giemsa stain at 1:30 dilution and cells were counted using a hemacytometer Neubauer under the light microscope. The leukocyte differential calculation was performed in peripheral blood spots stained by Merck Giemsa, giving the Neutrophils quantity of differential neutrophils and the mononuclear quantity of differential lymphocytes, eosinophile and monocyte. Hematocrite percent (Ht%) was shortly computed after act of collecting representative samples by placing fresh blood in glass capillary tubes and centrifuged in a microhematocrit centrifuge for 5 min at 10,000 rpm (Hettich, Germany), afterward, taking of dimensions the packed cell capacity. Hematocrite explanation was carried out with the usage of a microhematocrit reader. Hemoglobin levels (Hb mg/l) were resulted colorimetrically of cyanomethemoglobin by measuring the formation [9]. Erythrocytes Indices (M.C.H. or Mean Corpuscular Hemoglobin, M.C.H.C. or Mean Cell Hemoglobin Concentration and M.C.V. or Mean Corpuscular Volume) were accounted from RBC, Ht, and Hb [9, 10].

### Statistical analyses

To observation of significant differences to appraise, the effect of diazinon on blood parameters of silver carp was used an analysis of variance (ANOVA) with Duncan Post Hoc. Pearson coefficients of correlation (*r*) were calculated between half-lethal concentration of diazinon and blood parameters to examine coalition between bioaccumulation and its impacts. To determine the unity between diazinon concentration and blood parameters were utilized multiple regressions. Data were analyzed statistically at *p* < 0.05 by SPSS software version 16.

### Ethical approval

This work was approved by ethical committee of GAU University numbered 6177515-5. To minimize suffering of the fish, all animals were exposed with clove essence, low dose for anesthesia; hypothermia prior to euthanasia and eventually spinal cord dislocation for euthanasia.

## Results and discussion

### Hematological parameters

In this study, we surveyed effects of 50 % LC50 diazinon pesticide on a series of hematological, biochemical and Immunological parameters of silver carp for 96 h.

**Fig. 1:**
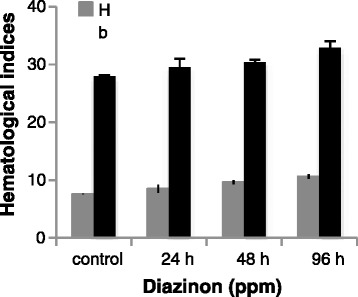
Haematocrite and Hemoglobin changes of silver carp during exposure to LC_50–96h_ diazinon

Hematocrit content significantly elevated after subchronic exposure, exhibiting the importance of the route of pollution (Fig. 1). Also, Ht and Hb levels significantly increased that was similar with results of Ahmad [11] on Cyprinus carpio in exposed to diazinon but was conversed with the findings Shaluie et al. [12] on silver carp exposed to Nanocid. Also Banaee et al. [13] and Anees [14], respectively, to study on Cyprinus carpio and Bloch (*Channa punctatus*) concluded that Ht and Hb levels significantly increased in exposed to diazinon. In another studies, Oimoek Köprücü et al. [15] found out that Ht and Hb levels increased in fingerling European catfish (*Silurus glanis L*.) exposed to diazinon. Observed results were agreement with results of Chowdhury et al. [16], who stated an elevation of blood hemoglobin and hematocrit happen in exposed to environmental hypoxia and chronic doses of waterborne metals that elevate blood oxygen carrying capacity caused to disorder of gas exchange.

**Fig. 2:**
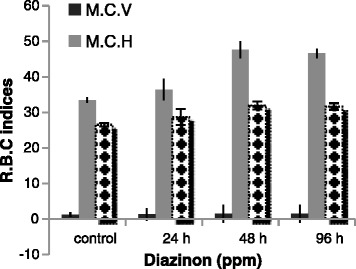
M.C.V., M.C.H. and M.C.H.C. change of silver carp during exposure to LC_50–96h_ dazinon

Our findings showed that MCV and MCH levels increased in fish exposed to 50 % LC50 diazinon at 48 h but MCHC level increased at 96 h (Fig. 2). Results of the study were similar with the findings of Banaee et al. [13] about MCV and MCH. Shaluie et al. [12] to survey on silver carp exposed to Nanocid expressed that MCH and MCHC levels raised. Also, Mohammad Nejad et al. [17] and Anees [14] to studying on *Rutilus frisii kutum* and Bloch (*Channa punctatus*) in exposed to diazinon found out that MCH, MCV and MCHC levels increased that were agreement with our study.

In the present study, WBC level of silver carp blood significantly increased in exposed to diazinon. Whereas, Banaee et al. [13] and Mohammad Nejad et al. [17] obtained opposite results to this finding. The number of red blood cells (RBC) was less sensitive toward diazinon exposure so that the RBC count did not significantly changed in fish. This finding was not similar with Mohammad Nejad et al. [17], Banaee et al. [13], Khoshbavar Rostami and Soltani [18] and Anees’s studies [14] on *Rutilus frisii kutum*, *Cyprinus carpio*, *Acipenser nudiventris* and Bloch (*Channa punctatus*) in exposed to diazinon. Plenty of WBC provides an exhibition of fish health and a high WBC number may exhibit a subclinical infection.

The correlation between diazinon with all parameters statistically examined by analyzing the data obtained during the diazinon exposed for 96 h. In this concentration (LC_50–96h_) Ht, MCV, MCH and MCHC levels indicated significant positive correlation (*p* < 0.01) and WBC, RBC and Hb levels did not showed significant correlation with diazinon exposure.

### Biochemical analysis

**Fig. 3:**
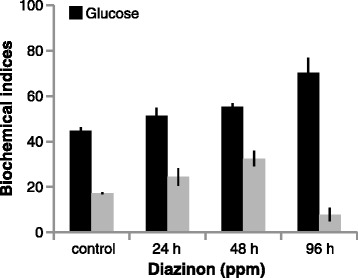
Cortisol and Glucose change of silver carp during exposure to diazinon

Glucose level significantly (*p* < 0.05) increased in 50 % LC_50_ concentration of diazinon in comparison of control group (Fig. 3) and showed significant positive correlation (*p* < 0.01) in exposure to diazinon.

It has been stated that the raised blood glucose is usually perceived in fish under unfavorable situations and it helps the animal by readying energy substrates to vital organs to afford with the increased energy demand [13]. Also, increased of blood glucose level was vastly used as a secondary marker of a stress response [19]. Increased in blood glucose levels have been reported in Silver carp [12] and *Cyprinus carpio* [13], after exposure to copper sulfate and diazinon, respectively, that was comparable to our findings of glucose on silver carp.

**Fig. 4:**
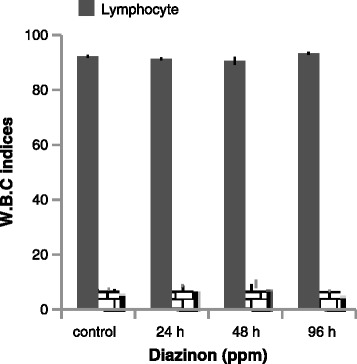
Lymphocyte and Neutrophil change of silver carp during exposure to diazinon

Blood cortisol is an important corticosteroid hormone in fish and may have a meaningful effect on its dynamics [20]. In the present study, cortisol level in concentration of 50 % LC50 for 48 h significantly increased toward control (Fig. 3) and did not show significant correlation in exposure to diazinon. The blood serum cortisol results were indicated in (Fig. 4), revealed a significant increasing at 48 h in the exposed fish. Bakhshwan et al. [21] and Shaluie et al. [12] obtained similar results to studying on Clarias gariepinus and silver carp in exposed to diazinon and Nanocid, respectively. The above data may be stated by the activation hypothalamo-pituitary- inter renal axis with their discharge of steroid cortisol in blood circuit due to stress [22]. In addition, it could be ascribed to the raise in osmotic water-influx, which may reason a cortisol promotion, to mend the hydromineral balance [21].

### Immunological indices

Lymphocyte level was significantly increased in exposed to diazinon for 96 h and neutrophil level was significantly increased for 48 h (*p* < 0.05) (Fig. 4). Eosinophiles and monocyte did not show significant correlation in 50 % LC50 concentration.

Results of current study showed that there were significant increases in lymphocyte and neutrophil counts. Hedayati and Jahanbakhshi [23] reported increasing in neutrophil and decreasing in lymphocyte to study on the great sturgeon Huso huso in exposed to diesel oil. In addition, Soltani and Khoshbavar Rostami [24] stated elevation neutrophil count and decreasing of lymphocyte count in *Acipenser guldenstadti*. In other studies, Svoboda et al. [25] and Banaee et al. [13] observed neutrophil count increased but lymphocyte count decreased. It is believed that neutrophils have phagocytic activity, which might describe their elevated percentage during infectious status.

To determine the relationship between diazinon concentration with hematological, biochemical and immunological activity were utilized curve estimation regressions data. Ht, MCV, MCH, MCHC, Lymphocyte and Glucose levels in fish exposed to diazinon indicated significant linear regression (*p* < 0.01) Y = a ± bX.

## Conclusion

The present results indicated that diazinon can cause serious hematological and biochemical alterations in silver carp at the physiological level. Finally, the results got in this study exactly indicated under experimental conditions, blood parameters were sensitive to different prospect of diazinon exposure. Hematological and biochemical properties of this fish exposed to diazinon are poorly understood and there is not adequate knowledge concerning the metabolism of reference toxicants. More knowledge of these activities in fish is necessary before they can be employed as biochemical indicators of stress due to pollutions.
